# Enhancing the Shock Response Performance of Micromachined Silicon Resonant Accelerometers by Electrostatic Active Damping Control

**DOI:** 10.3390/mi12121548

**Published:** 2021-12-12

**Authors:** Libin Huang, Kai Jiang, Peng Wang, Meimei Zhang, Xukai Ding, Hongsheng Li

**Affiliations:** 1 School of Instrument Science and Engineering, Southeast University, Nanjing 210096, China; jiangkai142857@163.com (K.J.); 220203615@seu.edu.cn (P.W.); 220193328@seu.edu.cn (M.Z.); ding.xk@seu.edu.cn (X.D.); hsli@seu.edu.cn (H.L.); 2 Key Laboratory of Micro-Inertial Instruments and Advanced Navigation Technology, Ministry of Education, Nanjing 210096, China

**Keywords:** resonant accelerometer, electrostatic active damping control, shock response performance

## Abstract

This paper presents a micromachined silicon resonant accelerometer based on electrostatic active damping control, which can improve the shock response performance of the accelerometer. In the accelerometer, an electrostatic active damping structure and damping control circuit are designed to improve the equivalent damping coefficient of the system. System-level Simulink modeling and simulation of the accelerometer with an electrostatic active damping closed-loop control link were carried out. The simulation results indicate that the system can quickly return to normal output without an obvious vibration process after the shock. The fabricated and packaged accelerometer was connected to an external test circuit for shock performance testing. The stabilization time of the accelerometer after a 100 g, 3–5 ms half-sine shock was reduced from 19.8 to 5.6 s through use of the damping control. Furthermore, the change in deviation before and after the shock without damping control was 0.8197 mg, whereas it was 0.1715 mg with damping control. The experimental results demonstrate that the electrostatic active damping control can effectively improve the dynamic performance of the micromachined silicon resonant accelerometer.

## 1. Introduction

A micromachined silicon resonant accelerometer is a micro-inertial device that uses the force–frequency characteristics of the resonator to sense acceleration, and its output is a quasi-digital signal in the form of frequency, which is easy to detect and digitally integrate. Micromachined silicon resonant accelerometers have the advantages of strong anti-interference capability, high resolution and sensitivity, wide dynamic range, good stability, and so on, and have shown good prospects for applications in navigation, automation, robotics, and other fields [1].

In the practical application of micromachined silicon resonant accelerometers, the mechanical environment of the application scenario is complex and variable, often featuring shock loads with multiple frequency components. Moreover, micromachined silicon resonant accelerometers often need to be packaged in a highly vacuumed chamber to obtain a very high quality factor, which greatly prolongs their stabilization time after shock loads and makes the resonant modes of sensitive structures more easily excited, which affects the actual performance of the accelerometer [2,3,4].

At present, there is little research on the shock response performance of micromachined silicon resonant accelerometers; most studies have been conducted on other MEMS accelerometers. In 2009, Yang, Z.X. et al. conducted a study on the shock response performance of piezoresistive MEMS accelerometers considering the material properties of the key sensitive structure beam of piezoresistive MEMS accelerometers, investigating the relationship between the doping concentration of silicon material, ambient temperature, and the damping coefficient of its beam structure [5]. In 2010, Y. Meyer et al. introduced a piezoelectric material-based active vibration isolation module to the proof mass of the accelerometer and controlled the active vibration isolation effect of the piezoelectric material by sensing the external shock vibration load effect with an appropriate control loop. The active vibration isolation effect of the material can avoid the excitation of the sensitive structure resonant mode by the shock signal and improve the MEMS accelerometer shock response [6]. In 2011, M. Dienel et al. investigated the vacuum level, with respect to the shock response performance of MEMS vibration or acceleration sensors, in the context of the development of high vacuum packages for MEMS devices [7]. In 2016, C. Kavitha et al. studied the effect of damping on the dynamic performance of MEMS capacitive accelerometers and constructed an equivalent circuit model of the device, considering the thermoelastic and squeeze film damping effects. They determined that the device has a better transient response when the medium used is helium [8].

All of these studies have improved the shock response performance of MEMS accelerometers by changing the type of medium in which the device is located, optimizing the structural and material properties, and introducing vibration isolation mechanisms. However, for micromachined silicon resonant accelerometers, the sensitive silicon structure is packaged in a high vacuum sealed cavity, which makes it hard to change the device medium and even impossible to change the material properties [9]. Furthermore, the introduction of a vibration isolation mechanism requires exploring a new processing or packaging process, for which the associated research period is long.

In this paper, a micromachined silicon resonant accelerometer based on an electrostatic active damping control is designed without modifying the main structural parameters of the original device. Closed-loop control is used to apply a suitable control force on the proof mass, in order to increase the system damping equivalently, thereby improving the shock response performance of the accelerometer.

## 2. Shock Response Analysis

**Figure 1:**
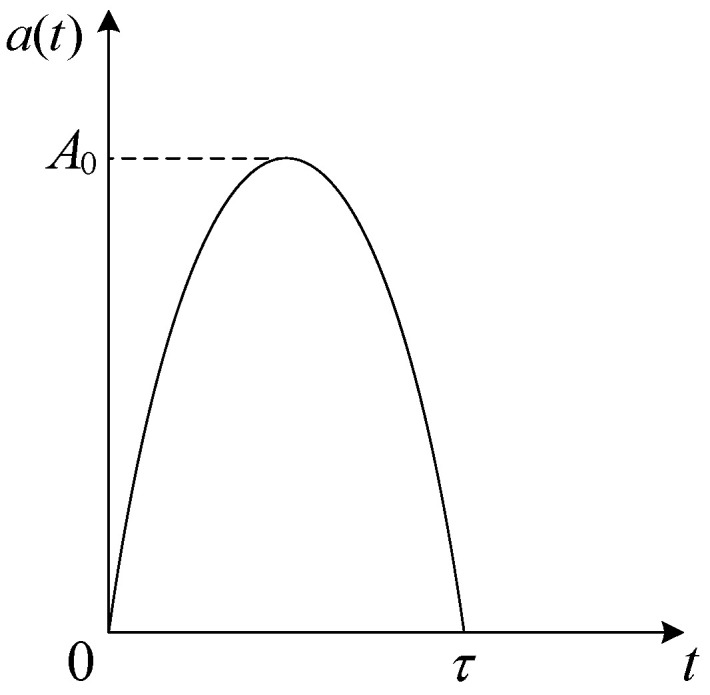
Half-sine shock load.

The complex shock environment can be conveniently approximated by a series of simple shock pulses in laboratory tests [10]. In the analysis of shock performance, half-sine shocks are usually used as a simplified model of the shock signal for analysis. The schematic diagram of an acceleration load is shown in Figure 1, and the function is [11]:

(1) $$a(t)=\left\{ \begin{array}{ll} A_{0}\sin\omega_{0}t & 0\leq t\leq \tau \\ 0 & t>\tau \end{array}\right.,$$

where $\omega_{0}=\frac{\pi }{\tau }$, *τ* is the duration of the acceleration load, and $A_{0}$ is the peak acceleration.

The basic mechanical model of a micromachined silicon resonant accelerometer is a mass-spring-damper second-order system. The differential equation of motion under a half-sine shock acceleration load can be obtained as [12,13]:

(2) $$m\frac{d^{2}x(t)}{dt}+c\frac{dx(t)}{dt}+kx(t)=F(t)=ma(t),$$

where m is the mass of the proof mass, c is the equivalent damping coefficient of the proof mass, k is the equivalent stiffness of the proof mass, and x(t) is set to be the displacement vector of the proof mass relative to the base.

This differential equation can be solved as:

(3) $$x(t)=\left\{ \begin{array}{ll} \frac{A_{0}e^{-\zeta \omega_{n}t}}{\omega_{d}}\cdot \{\frac{2\zeta \omega_{0}\omega_{n}\omega_{d}\left[\cos(\omega_{d}t)-e^{\zeta \omega_{n}t}\cos(\omega_{0}t)\right]}{\omega_{0}^{4}-2\omega_{0}^{2}(\omega_{d}^{2}-\zeta^{2}\omega_{n}^{2})+{\left(\omega_{d}^{2}+\zeta^{2}\omega_{n}^{2}\right)}^{2}}+ & \\ \frac{\left(\omega_{d}^{3}+\zeta^{2}\omega_{n}^{2}\omega_{d}-\omega_{0}^{2}\omega_{d})e^{\zeta \omega_{n}t}\sin(\omega_{0}t)+(\omega_{0}^{3}+\zeta^{2}\omega_{n}^{2}\omega_{0}-\omega_{0}\omega_{d}^{2})\sin(\omega_{d}t\right)}{\omega_{0}^{4}-2\omega_{0}^{2}(\omega_{d}^{2}-\zeta^{2}\omega_{n}^{2})+{\left(\omega_{d}^{2}+\zeta^{2}\omega_{n}^{2}\right)}^{2}}\} & 0\leq t\leq \tau \\ \frac{A_{0}e^{-\zeta \omega_{n}t}}{\omega_{d}}\cdot \{\frac{2\zeta \omega_{0}\omega_{n}\omega_{d}\left\{\cos(\omega_{d}t)+e^{\zeta \omega_{n}\tau }\cos[\omega_{0}(t-\tau )]\right\}}{\omega_{0}^{4}-2\omega_{0}^{2}(\omega_{d}^{2}-\zeta^{2}\omega_{n}^{2})+{\left(\omega_{d}^{2}+\zeta^{2}\omega_{n}^{2}\right)}^{2}}+ & \\ \frac{(\omega_{0}^{3}+\zeta^{2}\omega_{n}^{2}\omega_{0}-\omega_{0}\omega_{d}^{2})\cdot \left\{\sin(\omega_{d}t)+e^{\zeta \omega_{n}\tau }\sin[\omega_{0}(t-\tau )]\right\}}{\omega_{0}^{4}-2\omega_{0}^{2}(\omega_{d}^{2}-\zeta^{2}\omega_{n}^{2})+{\left(\omega_{d}^{2}+\zeta^{2}\omega_{n}^{2}\right)}^{2}}\} & t>\tau \end{array}\right.$$

where $\omega_{n}=\sqrt{\frac{k}{m}}$ is the undamped free vibration angular frequency of the system, $\zeta =\frac{c}{2\sqrt{km}}$ is the damping ratio of the system, and $\omega_{d}=\omega_{n}\sqrt{1-\zeta^{2}}$ is the angular frequency of the damped vibration of the system.

According to Equation (3), both the response amplitude and the time constant of the attenuation process after the action of the shock signal are related to the undamped free vibration angular frequency $\omega_{n}$ and the damping ratio ζ. For the proof mass of the silicon structure, which is packaged in a high vacuum environment, the system damping coefficient is extremely low, which causes the oscillation process of the proof mass after the shock load to have a large time constant. Thus, the system decays slowly over a long time, affecting the normal response of the system to acceleration after the shock load. Therefore, when the undamped free vibration angular frequency $\omega_{n}$ is constant, the value of the damping coefficient will directly affect the response capability of the system to the shock load, and increasing the equivalent damping of the system will reduce the response amplitude and stabilization time after the shock load.

## 3. Micromachined Silicon Resonant Accelerometer Based on Electrostatic Active Damping Control

### 3.1. Basic Principles of Electrostatic Active Damping Control

The proposed accelerometer adopts a system damping control method based on electrostatic active damping control, using a closed-loop control to apply a suitable force to the proof mass to equivalently increase the system damping. Consequently, the time constant of the oscillation process of the silicon structure’s proof mass is reduced both during and after the shock load, and the system’s response to the shock signal is improved.

**Figure 2:**
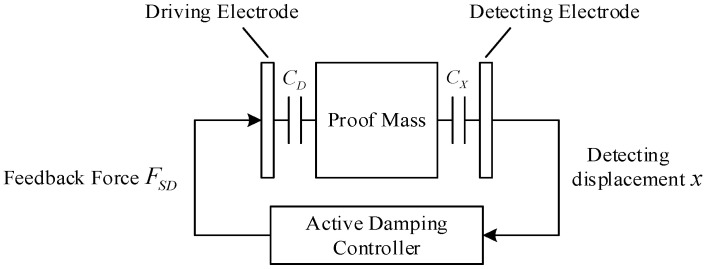
Block diagram of the principle of the electrostatic active damping control.

To match the existing structure, this feedback control force is applied by an electrostatic force. The block diagram of the principle of the electrostatic active damping control is shown in Figure 2.

When the proof mass is displaced by an external acceleration, the detecting electrode captures the displacement signal and changes it into a voltage signal. Then, the active damping controller processes the voltage signal and generates a feedback voltage signal to act on the driving electrode [14,15], which transforms the signal into an electrostatic force $F_{SD}$. When the feedback force $F_{SD}$ acts on the proof mass, the kinetic equation of the proof mass is:

(4) $$m\frac{d^{2}x(t)}{dt}+c\frac{dx(t)}{dt}+kx(t)=F(t)-F_{SD}.$$

If the relationship between the feedback force and the displacement of the proof mass is in the following form:

(5) $$F_{SD}=c^{\prime }\frac{dx(t)}{dt},$$

the equivalent damping of the system will change from c to $c+c^{\prime }$, where $c^{\prime }$ is the increase in the damping coefficient of the system by the damping controller. The above equation shows that the introduction of feedback control with differential property to the proof mass will equivalently increase the damping of the system.

The implementation of the electrostatic active damping control consists of two parts: structural and circuit alteration. On the one hand, active damping detecting and driving combs are added to the existing structure, in order to detect the displacement of the proof mass and apply the electrostatic drive force to the proof mass, respectively. On the other hand, the active damping control circuit needs to be designed to realize the relationship in Equation (5) between the input proof mass displacement and the output feedback electrostatic force.

### 3.2. Structural Design

**Figure 3:**
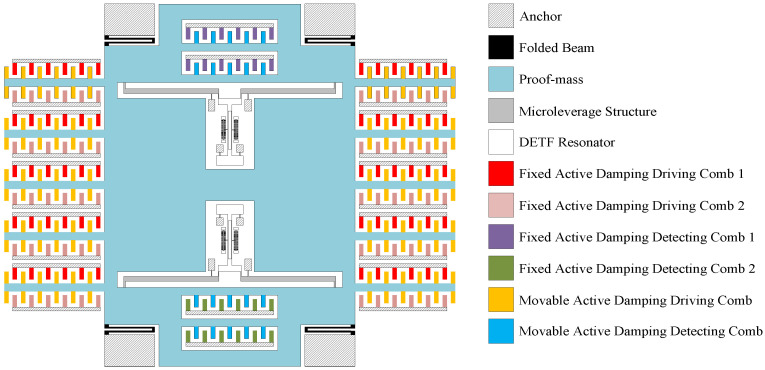
Overall structure of a micromachined silicon resonant accelerometer based on the closed-loop control of electrostatic active damping.

The schematic diagram of the overall structure of the micromachined silicon resonant accelerometer based on the closed-loop control of electrostatic active damping is shown in Figure 3.

In this structure, active damping driving and detecting combs are added to the existing accelerometer proof mass [16]. Fixed active damping driving combs 1, fixed active damping driving combs 2, fixed active damping detecting combs 1, and fixed active damping detecting combs 2, as shown in Figure 3, are connected with each other through the bottom wiring and, therefore, can be considered the same point in the logic of the electrical connection. Both the movable active damping driving comb and the movable active damping detecting comb use the proof mass itself as the comb frame, in order to convert the displacement into the change in the overlap length of the detecting comb and the control voltage into the electrostatic driving force of the proof mass. This structure thereby realizes the electrostatic active damping closed-loop control. The specific structural dimensions associated with the electrostatic active damping control are provided in Table 1. During the subsequent simulation analysis of this structure, the comb structure is aggregated and equated to facilitate the simulation analysis.

**Figure 4:**
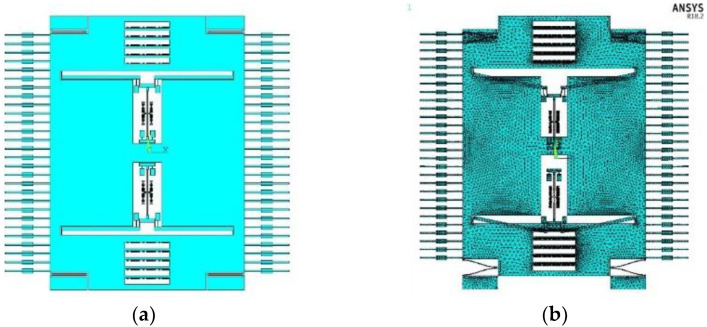
(**a**) The structural model and (**b**) the operating mode of the proof mass.

Structural finite element analysis was carried out using the ANSYS 18.2 software, and the model was established using SOLID 95 elements. The overall structure, under the action of the shock load with an amplitude of 100 g and a duration of 3 or 5 ms, satisfied the silicon material stress condition in all directions. No collision would occur between the parts of the structure, and no structural failure occurred. The block Lanczos method was used to extract the first 30 modes, and the first mode of the proof mass was the operating mode. The first modal resonant frequency was 1707.5 Hz, and the established finite element model and modal simulation results are shown in Figure 4.

### 3.3. Damping Control Circuit

#### 3.3.1. Calculation of System Parameters

**Figure 5:**
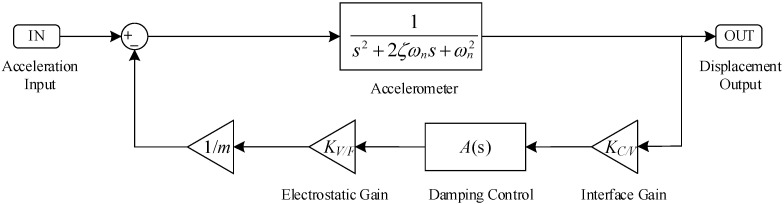
Block diagram of the electrostatic active damping control system for the micromachined silicon resonant accelerometer.

The overall block diagram of the micromachined silicon resonant accelerometer system, including the damping control, is shown in Figure 5, where the interface circuit implements the C/V conversion of the displacement signal with a gain factor of $K_{C/V}$. The drive electrode exerts an electrostatic force on the proof mass under the action of the control voltage with a gain factor of $K_{V/F}$, and the transfer function of the damping control link is defined as A(s).

According to Figure 5, the transfer function of the closed-loop system of the micromachined silicon resonant accelerometer with active damping control can be expressed as:

(6) $$H(s)=\frac{1}{s^{2}+2\zeta \omega_{n}s+\omega_{n}^{2}+\frac{K_{C/V}K_{V/F}}{m}A(s)}.$$

The front-end interface in Figure 5 uses a ring diode-type capacitor detection circuit to realize the modulation and demodulation of the signal [17]. The external capacitors are 10 pF, the peak-to-peak value of the high-frequency carrier is 5 V, the conduction voltage drop of one diode is 0.34 V, and the amplification of the operational amplifier is 20. Then, the gain between the front-end displacement detection and the output voltage $K_{C/V}$ was calculated as 1.376 × 10^6^.

When the DC drive voltage is 60 V and the dielectric constant ε is 8.85 × 10^−12^, from the accelerometer’s structural parameters listed in Table 1, we found that the driving force gain factor, $K_{V/F}$, in the bilateral electrostatic comb drive method is 1.03 × 10^−4^. 

With the vacuum degree of the packaging and the structural dimension parameters, the viscous damping coefficient c of the system can be calculated as 56.337 × 10^−6^ N/(m/s). The proof mass m can be calculated as 3.2 × 10^−6^ kg from the structural parameters of the proof mass, and the first-order resonant frequency of the proof mass is 1.7075 kHz. The damping ratio of the system can be calculated as:

(7) $$\zeta =\frac{c}{2\sqrt{km}}=\frac{c}{2m\omega_{n}}=\frac{56.337\times 10^{-6}}{2\times 3.2\times 10^{-6}\times 2\pi \times 1707.5}=8.1833\times 10^{-4}.$$

Apparently, the damping ratio of the system is extremely low and will lead to significant underdamping characteristics; therefore, the system will not respond well to shock loads. When the gain coefficient of the damping control link is A, the increase in the damping coefficient $c^{\prime }$ of the system is $K_{C/V}\cdot A\cdot K_{V/F}$. Control theory states that, for a typical second-order system, when the damping ratio is 0.6–0.8, the system obtains a better comprehensive performance and does not have obvious resonance peaks; therefore, the control target of the system is taken as the system’s equivalent damping ratio reaches 0.7 [18]. Then, the system damping coefficient should be:

(8) $$c^{\prime \prime }=0.7\times 2\times m\times \omega_{n}=4.806\times 10^{-2}\;N/(m/s).$$

The increase in the damping coefficient of the system by the damping controller $c^{\prime }$ is about 4.8 × 10^−2^ N/(m/s), which gives a gain coefficient of the damping control link A of about 4 × 10^−4^.

#### 3.3.2. Damping Control Circuit Design

The damping control circuit is the core control unit of the accelerometer’s electrostatic active damping closed-loop control. The design of the damping control circuit directly affects the control capability of the electrostatic active damping and the overall performance of the system. In the frequency domain, the effects of the damping control circuit on the optimization of the accelerometer’s shock response performance are mainly reflected in the improvement of its equivalent damping. In the time domain, the damping control circuit mainly achieves weakening of the signal components at the resonant frequency of the proof mass, whereas the frequency range above the resonance point should be further attenuated, thus making the damping control circuit much less sensitive to high-frequency noise signals. 

**Figure 6:**
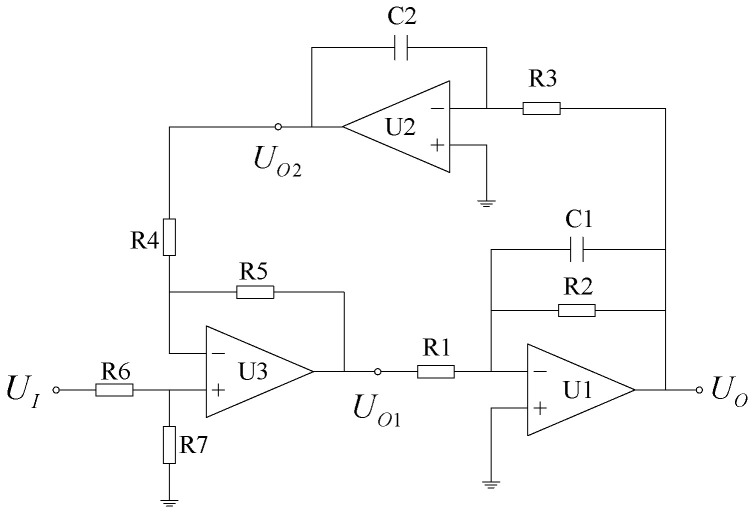
Damping control circuit.

Based on the above analysis, the designed damping control circuit schematic is shown in Figure 6. The op-amp U1 implements the integration operation of the signal, op-amp U2 implements the first-order low-pass filtering operation of the signal, and op-amp U3 implements the summation and inverse operation of the two input signals.

According to the integrated op-amp virtual short and virtual break equivalent principle, if $R_{4}=R_{5}=R_{6}=R_{7}$, the damping control circuit’s transfer function can be expressed as:

(9) $$A(s)=\frac{U_{O}}{U_{I}}=\frac{\frac{1}{R_{1}C_{1}}s}{s^{2}+\frac{1}{R_{2}C_{1}}s+\frac{1}{R_{1}C_{1}R_{3}C_{2}}}.$$

The following parametric relationships are assumed as:

(10) $$\{\begin{array}{c} A=R_{3}C_{2}\\ \omega =\sqrt{\frac{1}{R_{1}C_{1}R_{3}C_{2}}}\\ r=\frac{1}{2\omega R_{2}C_{1}} \end{array}.$$

Then, Equation (9) can be rewritten as:

(11) $$A(s)=\frac{U_{O}}{U_{I}}=\frac{A\omega^{2}s}{s^{2}+2r\omega s+\omega^{2}}\prime$$

where A characterizes the differential time constant of the damping control circuit, which is also the gain coefficient of the damping control link; ω characterizes the turning angle frequency of the damping control circuit; and r characterizes the equivalent damping ratio of the damping control circuit.

Equation (11) shows that the damping control circuit is equivalent to a series connection of a pure differential link and a typical second-order system with a time constant of A, thus making the pure differential link increase by two complex poles. According to control theory, the amplitude–frequency characteristic curve describing the differential link will add a downward turning point to the original linear rise, where the angular frequency at the turning point is ω. The amplitude–frequency characteristic before the turning point will rise at a rate of 20 dB per decade and will fall at a rate of −20 dB per decade after the turning point. Hence, this will result in realizing the differential operation of signals below the turning frequency range and the attenuation characteristics of signals above the turning frequency.

Adding the damping control circuit’s transfer function from Equation (11) to Equation (6), the micromachined silicon resonant accelerometer closed-loop control system’s transfer function is:

(12) $$H(s)=\frac{\left(s^{2}+2r\omega s+\omega^{2}\right)}{(s^{2}+2\zeta \omega_{n}s+\omega_{n}^{2})(s^{2}+2r\omega s+\omega^{2})+\frac{K_{V/F}K_{C/V}A\omega^{2}}{m}s}.$$

**Figure 7:**
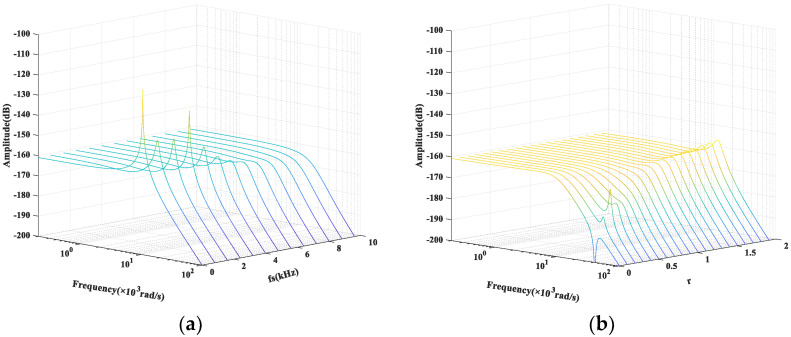
The system amplitude–frequency characteristic curve cluster: (**a**) when $f_{s}$ varies and (**b**) when r varies.

When r = 1, the damping control circuit is equivalent to a critical damping state. Let the damping control circuit turn frequency be $f_{s}$, such that $\omega =2\pi f_{s}$. The system amplitude–frequency characteristic curve cluster when $f_{S}$ varies is shown in Figure 7a. It can be seen, as the parameter $f_{s}$ gradually increases, that the system first shows obvious underdamping characteristics. Then, as the parameter $f_{s}$ continues to increase, the system resonance peak gradually decreases. Finally, the equivalent damping of the system is enhanced, which suppresses the system’s oscillation process and improves its shock performance. When $f_{s}$ is greater than 6.4 kHz, the system no longer has a significant resonance peak. If $f_{s}$ = 6.4 kHz (i.e., ω = 4 × 10^4^ rad/s), the amplitude–frequency characteristic curve cluster of the system when r takes different values is shown in Figure 7b. It is clear that the spike in the amplitude–frequency characteristic brought on by a lower r equivalent damping ratio will cause the system to be unstable, whereas a higher equivalent damping ratio r will weaken the damping control ability of the system and lead to a resonance peak. When r is around 0.7, the system can obtain comparatively ideal damping control performance. Considering the actual application scenario and circuit conditions, we finally take r = 0.6 and $f_{s}$ = 6.4 kHz to improve the damping control performance of the control loop.

**Figure 8:**
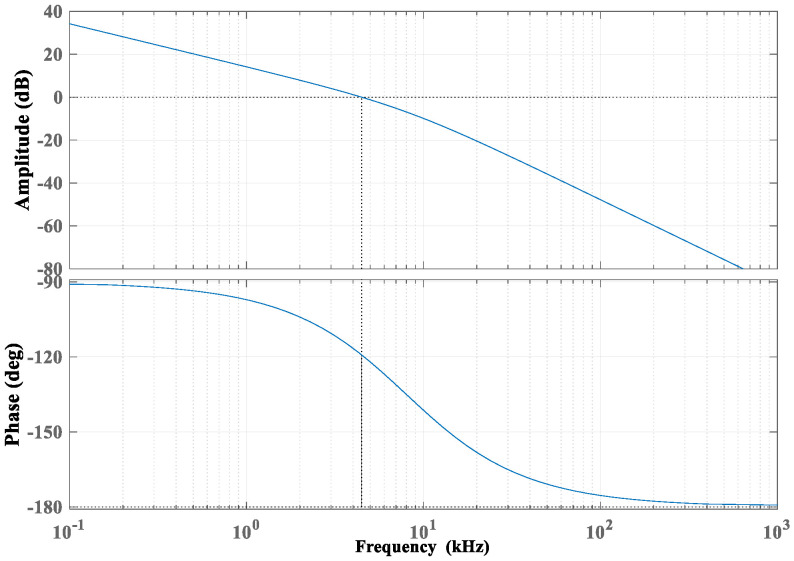
Open-loop Bode diagram of the damping control circuit.

The open-loop Bode diagram of the damping control circuit is shown in Figure 8.

From Figure 8, the phase margin of the damping control circuit is $60.8^{{}^{\circ}}$ and the amplitude margin is positive, which satisfies the strong stability condition of the system, such that the phase shift caused by the parasitic capacitance in the actual circuit does not affect the stability of the system.

#### 3.3.3. System Simulation and Analysis

**Figure 9:**
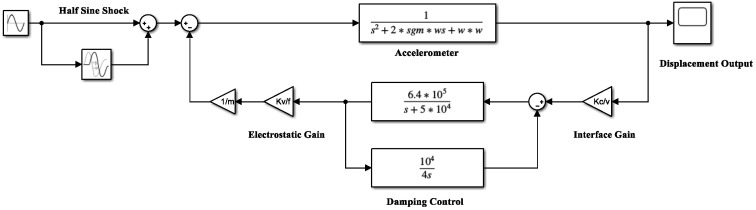
Simulink model of the electrostatic active damping closed-loop control.

A system-level Simulink modeling simulation of a micromachined silicon resonant accelerometer after the introduction of the proposed electrostatic active damping closed-loop control is shown in Figure 9, and the simulation parameters of the system are shown in Table 2.

**Figure 10:**
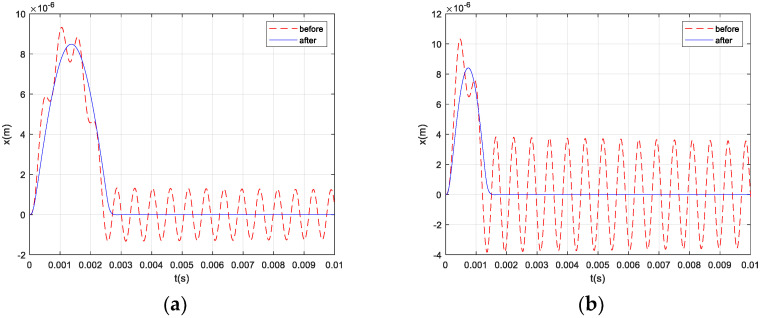

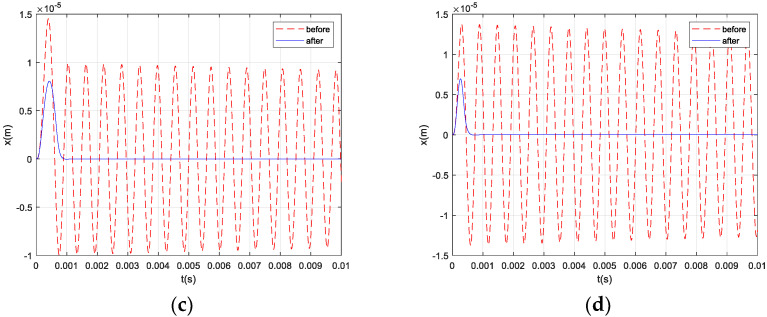
System shock responses before and after the introduction of the electrostatic active damping control: (**a**) *f*_0_ = 200 Hz; (**b**) *f*_0_ = 400 Hz; (**c**) *f*_0_ = 800 Hz; and (**d**) *f*_0_ = 1600 Hz.

According to the Simulink model shown in Figure 9, half-sine shock signals with an amplitude of 100 g and different frequencies *f*_0_ were applied to the system. The shock responses of the system before and after the introduction of the electrostatic active damping control are compared in Figure 10. It can be seen that, after the introduction of the electrostatic active damping control, the system behaved as a smoother half-sine signal output during the shock response, then quickly returned to normal output without an oscillation process after the shock load.

In summary, the dynamic performance of the micromachined silicon resonant accelerometer with the introduction of electrostatic active damping control was significantly promoted, due to the increase in the damping coefficient of the system.

## 4. Experiments

### 4.1. Fabricated Accelerometer Structure

**Figure 11:**
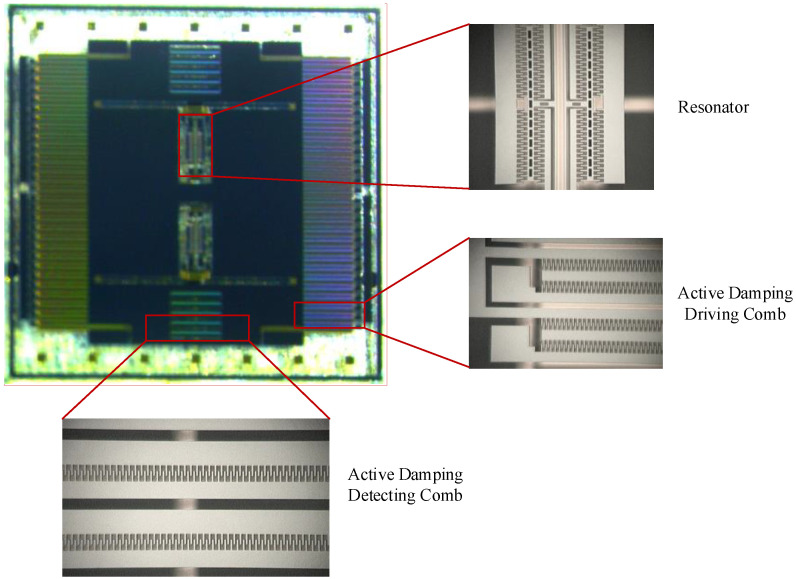
Photographs of the fabricated accelerometer structure.

The designed accelerometer structure was fabricated using the DDSOG (deep dry silicon on glass) body silicon process. Photographs of the micromachined silicon resonant accelerometer structure under the microscope are shown in Figure 11.

### 4.2. Test Circuit and Equipment

**Figure 12:**
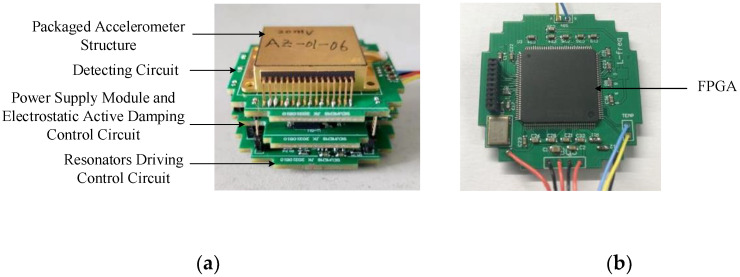
Photographs of the micromachined silicon resonant accelerometer: (**a**) packaged accelerometer structure and peripheral circuitry and (**b**) frequency measurement module.

In the performance test, the resulting sensitive structure was packaged in a high vacuum metal cavity, physically shown in Figure 12a. The PCB circuit board of the peripheral circuit consisted of three boards: an upper board with the packaged accelerometer structure and front-end detection circuit, a middle board with the power supply module and electrostatic active damping control circuit, and a lower board as the resonator driving control circuit, with electrical signals connected by pins between the two boards. The frequency signal of the resonator was connected to the frequency measurement module by a peripheral circuit, as shown in Figure 12b. The frequency difference data of the two resonators were calculated by an FPGA in the frequency measurement module and transferred to the upper computer.

**Figure 13:**
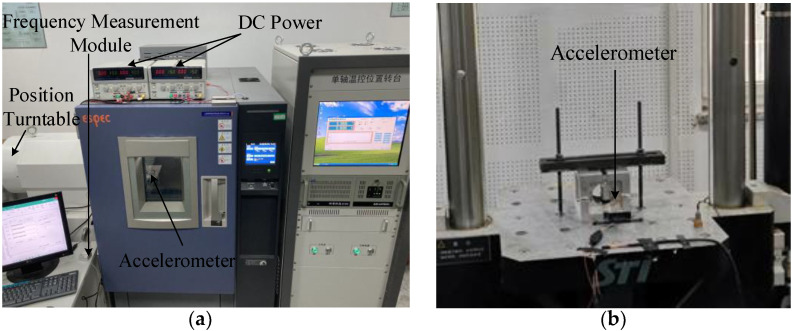
Photographs of experimental equipment: (**a**) single-axis temperature-controlled position turntable and (**b**) shock test apparatus.

The micromachined silicon resonant accelerometer test equipment mainly included DC power, a single-axis temperature-controlled position turntable, and a CL-100 STI shock test apparatus. Photographs showing some of the experimental equipment are provided in Figure 13. The ambient conditions during the experiment were room temperature and standard atmospheric pressure, and the power supply conditions were +15 V, −15 V, +60 V, and GND.

### 4.3. Shock Experiments

First, a micromachined silicon resonant accelerometer containing the electrostatic active damping closed-loop control was mounted on a single-axis temperature-controlled position turntable using fixture components. The scale factor K of the accelerometer was measured to be 144.2489 Hz/g.

Then, the micromachined silicon resonant accelerometer was mounted on the shock test apparatus using fixture components. When the accelerometer output was stable, the shock test apparatus was turned on and a single half-sine acceleration shock load with a peak acceleration of 100 g and duration of 3–5 ms was applied in the sensitive direction of the micromachined silicon resonant accelerometer, with and without the introduction of electrostatic active damping control. The output signal was sampled at a frequency of 1 kHz, and the sampling was stopped when the accelerometer output stabilized after the shock load.

After the shock action, if the output signal of the accelerometer did not exceed the fluctuation range of its static output, the device was considered to be at the end of the oscillation decay process. The time from the end of the shock action to the end of the decay process was recorded as the shock action stabilization time of the accelerometer.

By recording 10,000 sampling points before and after the shock and calculating the average of the frequency differences, the expression for the change in deviation before and after the shock was obtained as:

(13) $$\Delta B_{S}=\frac{\left|B_{before}-B_{after}\right|}{K},$$

where $B_{before}$ is the mean value of the frequency difference of the accelerometer before the shock and $B_{after}$ is the mean value of the frequency difference of the accelerometer after stabilization.

**Figure 14:**
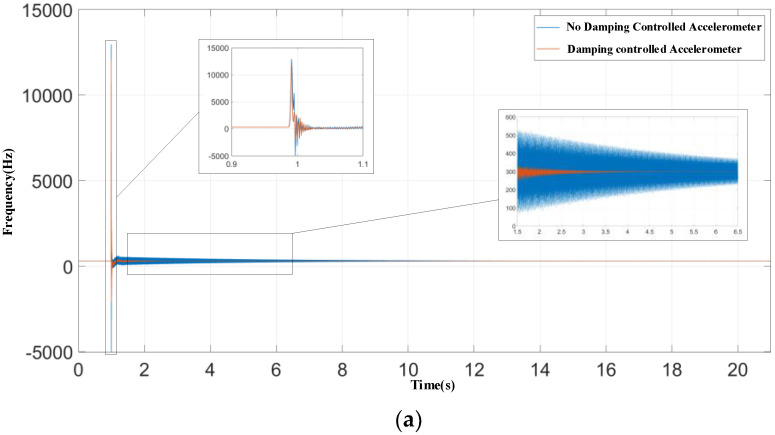

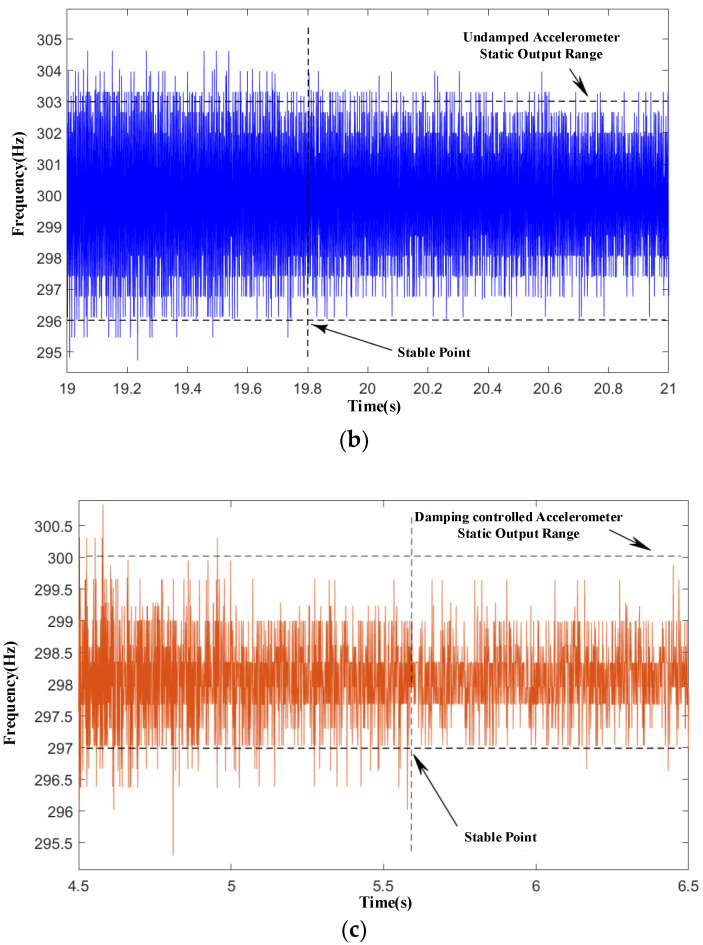
Experimental results: (**a**) Shock response results; (**b**) stabilization time with no damping control accelerometer; and (**c**) stabilization time with damping control accelerometer.

Half-sine shock experiments were performed on micromachined silicon resonant accelerometers with and without the electrostatic active damping control at ambient temperature, and the experimental results are shown in Figure 14 and Table 3.

The experimental results and calculation data show that, after the introduction of the electrostatic active damping control, the equivalent damping coefficient of the micromachined silicon resonant accelerometer was improved, with the stabilization time of the micromachined silicon resonant accelerometer after a 100 g, 3–5 ms half-sine shock being shortened from 19.8 to 5.6 s. Meanwhile, according to Equation (13), the change in deviation before and after the shock without damping control was 0.8197 mg, whereas it was 0.1715 mg with damping control. Obviously, the stability of the system under shock was improved.

## 5. Discussion

From the simulation and experimental results, the use of electrostatic active damping significantly improved the change in deviation and stabilization time of the micromachined silicon resonant accelerometer after shock. However, the electrostatic active damping control loop also operates when the accelerometer is in the non-shock state. This situation may bring disturbance to the accelerometer measurements, which can be bypassed by setting a displacement threshold. Enabling and disabling an active damping controller can then be achieved by judging the displacement threshold.

## 6. Conclusions

By analyzing the response of a micromachined silicon resonant accelerometer under the action of a half-sine shock load, it was found that the system damping coefficient directly affects its shock response performance. In order to increase the equivalent damping coefficient of the system, a drive comb and a detection comb for electrostatic active damping control were added to the original structure, and a damping feedback control circuit was designed to complete the design of the electrostatic active damping closed-loop control system. The actual test results demonstrated that the micromachined silicon resonant accelerometer with closed-loop electrostatic damping control designed in this paper has a scale factor of 144.2489 Hz/g. Under a half-sine shock of 100 g lasting 3–5 ms, both the change in deviation and the stabilization time were reduced. Thus, the proposed design improved the response performance of the micromachined silicon resonant accelerometer to shock loads and improved its adaptability in the shock mechanics environment. The micromachined silicon resonant accelerometer based on electrostatic active damping control technology has prospects for wide application in complex mechanical environments, which lays the foundation for its subsequent practical engineering application.

## Figures and Tables

**Table 1:** Structural dimensions associated with the electrostatic active damping control.

Parameters	Values
Proof mass equivalent area (m^2^)	22.89 × 10^−6^
Structure thickness (μm)	60
Number of active damping driving combs	6480
Active damping driving comb gap (μm)	4
Active damping driving comb length (μm)	30
Initial overlap length of active damping driving combs (μm)	15
Active damping driving comb width (μm)	4
Number of active damping detecting combs	1200
Active damping detecting comb gap (μm)	4
Active damping detecting comb length (μm)	30
Initial overlap length of active damping detecting combs (μm)	15
Active damping detecting comb width (μm)	4
Spacing between silicon structure and glass base (μm)	20

**Table 2:** Simulation parameters and their values.

Simulation Parameters	Values
Damping ratio ζ	8.1833 × 10^−4^
Undamped free vibration angular frequency $\omega_{n}$(rad/s)	10,681
Front-end interface gain $K_{C/V}$	1.376 × 10^6^
Damping control circuit gain A	4 × 10^−4^
Damping control circuit turning frequency $f_{S}$ (kHz)	6.4
Damping control circuit equivalent damping ratio r	0.6
DC drive voltage $V_{D}$(V)	60

**Table 3:** Experimental data of half-sine shock tests.

Index	No Damping Control	Damping Control
Stabilization time (s)	19.8	5.6
Average value of frequency difference before shock (Hz)	299.88205	298.1226832
Average value of frequency difference after stabilization (Hz)	300.00029	298.0979416
